# Safety during polypharmacy: A post-hoc analysis examining the safety profile of cariprazine with other antipsychotics in the cross-titration phase

**DOI:** 10.1192/j.eurpsy.2021.1424

**Published:** 2021-08-13

**Authors:** G. Vass, Á. Barabássy, I. Laszlovszky, B. Sebe, Z.B. Dombi, B. Szatmári, G. Németh

**Affiliations:** Medical Division, Gedeon Richter Plc., Budapest, Hungary

**Keywords:** Cariprazine, schizophrénia, polypharmacy, safety

## Abstract

**Introduction:**

Although monotherapy is preferable, in every day clinical practice polypharmacy is often unavoidable due to the need of treatment enhancement or cross-titration phases with shorter or longer overlaps of two or more drugs. However, administration of more than one drug treatment is often associated with more side effects.

**Objectives:**

The aim of the present post-hoc analysis was to examine treatment emergent adverse events (TEAEs) during co-administration of cariprazine with other antipsychotics.

**Methods:**

Treatment emergent adverse event data (TEAE) from a randomized, double-blind, parallel-group, active-controlled study (EudraCT Number: 2012-005485-36) in adult patients with schizophrenia having predominant negative symptoms was examined in the first two weeks of the double-blind treatment period, where gradual cross-titration occurred between cariprazine (3-6 mg/day) and other antipsychotics (including amisulpride, aripiprazole, fluphenazine, haloperidol, olanzapine, paliperidone, quetiapine, and sertindole). Thereafter, 24 weeks of cariprazine monotherapy followed.

**Results:**

During the cross-titration period, 17.83% of patients experienced at least one TEAE. The TEAEs were in line with the well-established safety data: nausea (2.61%), insomnia (2.17%), headache (2.17%), akathisia (1.74%) and restlessness (1.3%) were the most common. Most events were mild in severity (66.1% mild, 32.2% moderate, 1.7% severe (insomnia)).

**Conclusions:**

While not definitive, and limited by small sample size, the co-administration of cariprazine with other antipsychotics did not show an unexpected safety profile or overlapping toxicities. This is an important finding, if intermittent or longer co-administration of other antipsychotics are unavoidable with cariprazine treatment.

**Conflict of interest:**

Studies were funded by Gedeon Richter Plc. and Allergan Plc (prior to its acquisition by AbbVie). Dr Vass, Dr Barabássy, Dr Laszlovszky, Dr Sebe, Dombi, Dr Szatmári and Dr Németh are employees of Gedeon Richter Plc.

